# Prevalence of osteoradionecrosis demonstrated in 18F-FDG PET-CT of post-high-dose-radiotherapy head and neck cancer patients

**DOI:** 10.1186/1470-7330-14-S1-P3

**Published:** 2014-10-09

**Authors:** LI Sonoda, A Lakhani, S Ghosh-Ray

**Affiliations:** 1 Paul Strickland Scanner Centre, Mount Vernon Hospital, London, UK

## Introduction

Osteoradionecrosis (ORN) is one of the complications after high-dose-radiotherapy due to damage to normal bony tissues. ORN may occur years after radiotherapy and clinical manifestation may mimic disease recurrence. This study aimed to determine the incidence of ORN shown in 18F-FDG-PET-CT of post-radiotherapy head and neck cancer (HNC) patients.

## Methods

Retrospective analysis of 386 PET-CT scans of post-radiotherapy HNC was performed. Total dose of radiotherapy, time duration after radiotherapy and SUVmax were recorded. Final diagnosis was reached by biopsy, clinical and imaging follow-up.

## Results

Out of 386 scans, 41 cases demonstrated abnormal increased bony/cartilaginous uptake. Of which 22 were confirmed as residual local osseous involvement of pre-existing bony disease, 8 due to disease recurrence at new sites of bone/cartilage, 7 due to benign causes such as dental infection, and 4 due to ORN with no previous local osseous involvement. The incidence of ORN is 1.0% (4/136) of total PET-CT scans and 9.8% (4/41) of increased osseous FDG-uptake. Average occurrence of ORN was 6 months after radiotherapy (range 2 months to 3 years), all received over 60 Gray of radiation dose. Mean-SUVmax of ORN was 6.6, not significantly different from disease recurrence (mean-SUVmax=8.2,P=0.23).

## Conclusion

ORN is a rare complication after high-dose radiotherapy seen in PET-CT (1.0%), but when abnormal increased osseous FDG-uptake is seen within the previous radiotherapy field then ORN should be considered as a differential diagnosis (9.8% of increased osseous uptake). ORN may occur years after treatment and symptoms mimic disease recurrence. There is no significant difference in SUVmax values between ORN and recurrence.

